# Automatic medical encoding with SNOMED categories

**DOI:** 10.1186/1472-6947-8-S1-S6

**Published:** 2008-10-27

**Authors:** Patrick Ruch, Julien Gobeill, Christian Lovis, Antoine Geissbühler

**Affiliations:** 1 Medical Informatics Service, University and University Hospitals of Geneva, Geneva, Switzerland

## Abstract

**Background:**

In this paper, we describe the design and preliminary evaluation of a new type of tools to speed up the encoding of episodes of care using the SNOMED CT terminology.

**Methods:**

The proposed system can be used either as a search tool to browse the terminology or as a categorization tool to support automatic annotation of textual contents with SNOMED concepts. The general strategy is similar for both tools and is based on the fusion of two complementary retrieval strategies with thesaural resources. The first classification module uses a traditional vector-space retrieval engine which has been fine-tuned for the task, while the second classifier is based on regular variations of the term list. For evaluating the system, we use a sample of MEDLINE. SNOMED CT categories have been restricted to Medical Subject Headings (MeSH) using the SNOMED-MeSH mapping provided by the UMLS (version 2006).

**Results:**

Consistent with previous investigations applied on biomedical terminologies, our results show that performances of the hybrid system are significantly improved as compared to each single module. For top returned concepts, a precision at high ranks (P0) of more than 80% is observed. In addition, a manual and qualitative evaluation on a dozen of MEDLINE abstracts suggests that SNOMED CT could represent an improvement compared to existing medical terminologies such as MeSH.

**Conclusion:**

Although the precision of the SNOMED categorizer seems sufficient to help professional encoders, it is concluded that clinical benchmarks as well as usability studies are needed to assess the impact of our SNOMED encoding method in real settings.

**Availabilities:**

The system is available for research purposes on: .

## Introduction

**Figure 1:**
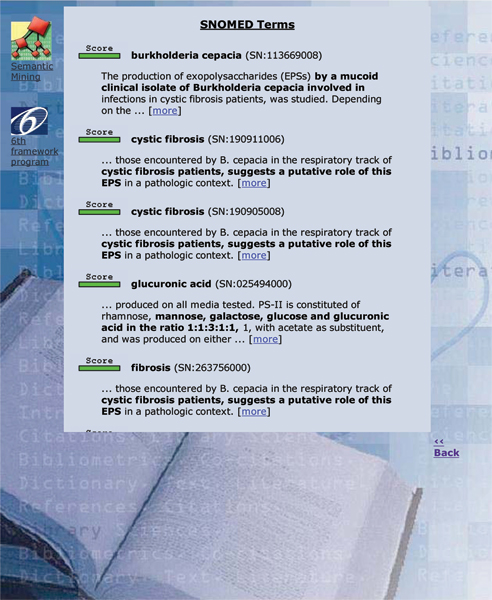
Output of the tool (categorization mode): these six categories are associated to the abstract shown in Figure 2. Some strings are duplicated because they refer to different concepts. The two top ranked concepts (*Burkholderia cepacia*; *Cystic fibrosis*;) are precisely those expected by the manual MeSH annotation of the article. Categories proposed at lower ranks (*glucuronic acid*;*fibrosis*) are irrelevant regarding the manual annotation performed by NLM (National Library of Medicine) librarians.

**Figure 2:**
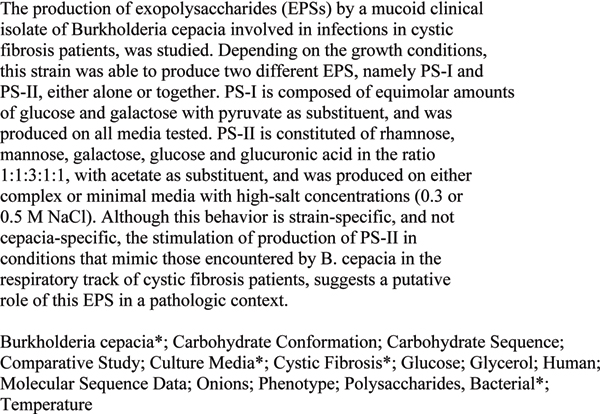
Citation with MeSH terms provided by professional indexers for PMID: 11506920.

**Figure 3:**
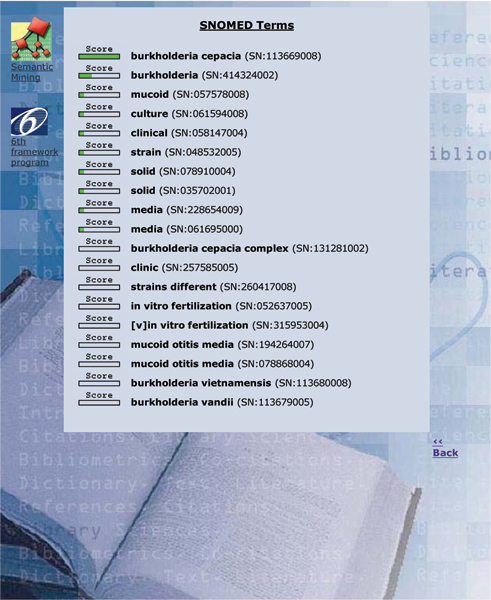
Output of the tool (browsing mode) with the query "Production in vitro, on different solid culture media, of two distinct exopolysaccharides by a mucoid clinical strain of Burkholderia cepacia": nineteen categories are displayed. The score associated with every predicted category drops after one to two terms, meaning that the quality of the association drops significantly.

SNOMED CT, the Systematized Nomenclature of Medicine Clinical Terms, represents an important advance in the field of biomedical terminological resources. Its broad coverage could be useful for several healthcare and management applications, in particular when deep semantic interoperability is of strategic importance: assisted generation of patient summaries, automatic processing of patient records, billing, fine-grained epidemiology studies, and text mining. However, this broad coverage can also be regarded as a major issue for SNOMED. Because it contains more than 380 000 concepts, with a total of about 800 000 descriptions or terms, the practical use of SNOMED will demand new types of tools to search and navigate intuitively in the term collection. The tool we describe in this paper is basically a terminology search tool. In a unique interface it combines: an interactive browser which returns a set of possible matches in the terminology given a short input text; a categorizer which attempts to assign a ranked set of categories given a long input text (such as the abstract of an article, a full-text article or a clinical narrative from a patient record); a passage retrieval tool, which associates each proposed SNOMED category with a short passage in the input document. Thus, the user can assess the quality of the automatic categorization by looking at the context, as explored for automatic curation [1] of the Swiss-Prot databases in molecular biology using the Gene Ontology, as well as for extraction GeneRiFs in ENTREZ-Gene [2]. The Gene Ontology categorizer (or GO categorizer) is available online , within the framework of the EAGL (Engine for Answering questions in Genomic Literature, ) project, while the SNOMED CT tool is normally in restricted access, available from the authors upon request. An example of the output of the tool is given in Figure 1 for the categorization mode. In that figure, the abstract in Figure 2 has been sent to the system: the association score which expresses the similarity between the input text and the category is represented by a scoring gauge, then the term of the category follows (the top returned concept in this document is *Burkholderia cepacia*), together with its SNOMED identifier, and finally a short passage (*The production of exopolysaccharides (EPSs) by a mucoid clinical isolate of Burkholderia cepacia involved in infections in cystic fibrosis patients, was studied. Depending on the growth conditions...*) which gives the context of the category in the input text is displayed. If only the title of the abstract is given to the system, i.e. when a short text is entered, the resulting output follows a browser mode: the ranked list returns more than fifty codes and no passage is given to support the proposed categories (Figure 3).

From a functional perspective, we can try to compare our tool with the well-known CLUE Browser. First, we observe that hierarchical visualization is not available in our tool, while it is an important functionality in the CLUE system, which can be seen as complementary to our system. On the opposite, the CLUE interface, as a strict browsing system, cannot accept as input a full document. In contrast, the string matching power of our system, which uses a retrieval-inspired conflation and normalization strategy – called stemming – clearly outperforms the CLUE browser regarding string approximation. Thus, the system handles most plural forms, morphological flexions and derivations as in *expressions*, *expresses*, *expressed*, *expressive*.... But the main advance of our categorizer is obviously located in its ranking power whose ranking strategies provide an optimal model of relevance as designed by large-scale user evaluation campaigns (e.g. [3]).

The remainder of this paper is organized as follows: the next section presents the data and metrics used in our experiments. Then, we present the methods used to perform the categorization task. Further, we propose a preliminary evaluation of the categorizer based on MEDLINE records, together with a qualitative evaluation, based on a few examples, which tries to exhibit SNOMED-specific features. Finally, we conclude on our experiments and suggest some future work to deliver an integrated and user-friendly system.

## Data and metrics

Because MEDLINE is indexed with Medical Subject Headings (MeSH) rather than with SNOMED codes, we need to transform MeSH terms, which are used to index MEDLINE records, into SNOMED terms. This translation is done thanks to the MeSH-SNOMED mapping table provided by the Unified Medical Language System. However, the translation process is not objective: it means that several MeSH terms cannot be mapped appropriately to SNOMED codes and vice versa. Thus, non medically-specific MeSH terms have usually no equivalent in SNOMED. Thus, technical categories methods, such as *Storage, data*, biological and pharmacological entities, such as *chaperonin *– which is mapped to *protein *– have often no appropriate equivalent in SNOMED. In all our experiments, we will assume that such an information loss is minor from a biomedical perspective and that the UMLS mapping between MeSH and SNOMED is sufficient to provide a basic evaluation of our SNOMED categorization tool. In order to assess our SNOMED categorizer, we apply the system on the Cystic Fibrosis (CF) collection [4,5]. The CF collection is a collection of 1239 MEDLINE citations. From this collection, 239 records were used for tuning our system and 1000 were used to evaluate our system. In the collection, MeSH items listed in MeSH fields are replaced by SNOMED codes. We demand that SNOMED codes are unique so that when two or more MeSH are mapped to the same SNOMED code, only one category is accepted. For each citation, we used the content of the abstract field as input for the categorizer. The average number of concepts per abstract in the collection is 12.3 and the average number of major terms is 2.8. From our MEDLINE records, only terms marked as major (with a star) are considered in our experiments. Following [6] and as it is usual with retrieval systems, the core measure for the evaluation is based on mean average precision. The top precision (interpolated *Precision*_*at Recall *= 0_), which is of major importance for a fully-automatic system, is also given.

## Methods

Two main modules constitute the skeleton of our system: the regular expression component, and the vector space component. Each of these basic classifiers uses known approaches to document retrieval. The former component uses tokens as indexing units and can take advantage of the thesaurus while the latter uses stems (i.e. strings such as *expression, expressed *are replaced by *express*). The first tool is based on a regular expression pattern matcher. Although such an approach is less used in modern information retrieval systems [7], it is expected to perform well when applied on relatively short documents such as SNOMED terms. It is to be observed that some SNOMED terms are particularly lengthy, at least when compared to MeSH terms: while most MeSH terms are shorter than six words, there are several terms SNOMED with more than a dozen of words. The second classifier is based on a vector-space engine [8]. This second tool is expected to provide high recall in contrast with the regular expression-based tool, which should privilege precision.

For a short introduction on automatic text categorization in MEDLINE, the reader is referred to the NLM's indexing initiative [9]; for a detailed presentation of our vector space engine and a comparison with state-of-the-art systems, including NLM's tools, see [3](in this joint evaluation between four retrieval systems, our engine showed competitive performances) [10]. For a complete overview and evaluation of our categorization system applied on Medical Subject Headings and on the Gene Ontology, see [11].

### SNOMED pre-processing

To be able to better associate SNOMED terms and textual entities, it is necessary to perform some pre-processing normalization steps. This includes removing meta-abbreviations, which are common in most terminological systems, such as NOS (Not otherwise specified), NEC (Not elsewhere classified), NOC (Not otherwise classifiable), or NFQ (Not further qualified). But we also need to handle and expand more than fifty SNOMED specific abbreviations, often inherited from Read codes, such as *ACOF; ADVA; AR; CFIO; CFSO; FB; FH; FHM; HFQ; LOC; MVNTA; MVTA*.

### Vector space system

The vector space (VS) module is based on a general information retrieval engine ; the engine, easyIR, which is available on the first author's homepage, with *tf.idf *(term frequency – inverse document frequency) weighting schema. In this study, it uses stems (Porter-like, with minor modifications) as indexing terms and an English stop word list. While stemming can be an important parameter in a text classification task, the impact of which is sometimes a matter of discussion [12], we did not notice any significant differences between the use of tokens and the use of stems. However, we noticed that a significant set of semantically related stems were not associated to a unique normalized string by the stemming procedure while they could have. Thus, the morpheme *immun *is found in 48 different stems. This suggests that more powerful conflation methods, based on morphemes [13], could have been used to enhance the recall of the current method, especially in multilingual contexts [14]. Altogether, we counted 72 402 unique stems in the SNOMED vocabulary.

A large part of this study was dedicated to tuning the VS engine, in which tf.idf weighting parameters were systematically evaluated. The conclusion is that cosine normalization was especially effective for our task. Consequently, in table 1, the top-4 weighting function uses cosine as normalization factor. We also observed that the *idf *factor, which was calculated on the whole SNOMED collection, performed well. This means that SNOMED is large enough to effectively underweight non-content words (such as *disease *or *syndrome*) which are very frequent in medical vocabularies but convey little meaning in the domain. Calculating the *idf *factor on a large collection of abstracts could have been investigated, but such a solution may have resulted in making the system more collection-dependent.

**Table 1:** Results for RegEx and (tf.idf) classifiers. weighting schemas. For the VS engine, tf.idf parameters are provided: the first triplet indicates the weighting applied to the "document collection", i.e. the concepts, while the second is for the "query collection", i.e. the abstracts.

System or parameters	Top precision	Mean average precision
RegEx	0.641	0.400

tf.idf (VS)		

lnc.atn	0.696	0.35525
anc.atn	0.691	0.3545
ltc.atn	0.75	0.33525
ltc.lnn	0.637	0.2775

### Regular expressions and synonyms

The regular expression (RegEx) pattern matcher is applied on the SNOMED concepts (376 212) augmented with its synonyms (the total includes 787 091 terms). In this module, text normalization is mainly performed by removing punctuation (e.g. hyphen, parenthesis...). The manually crafted transition network of the pattern-matcher is very simple, as it allows one insertion or one deletion within a SNOMED term, and ranks the proposed candidate terms based on these basic edit operations following a completion principle: the more tokens are recognized, the more the term is relevant. The system hashes the abstract into 6 token-long phrases and moves the window through the abstract. The same type of operations is allowed at the token level, so that the system is able to handle minor string variations, as for instance between *diarrhea *and *diarrhoea*. Interestingly, several morphological variations are directly provided by SNOMED descriptions.

Table 1 shows that the single RegEx (mean average precision = 0.4 and top-precision = 0.64) system performs better than the different settings tested for the vector space module, with tf.idf (term frequency-inverse document frequency) and length normalization (cosine...) factors, so that the thesaurus-powered pattern-matcher provides better results than the basic VS engine for SNOMED mapping. We use the (de facto) SMART standard representation in order to express these different parameters, cf. [15] for a detailed presentation. For each triplet provided in table 1, the first letter refers to the *term frequency*, the second refers to the *inverse document frequency *and the third letter refers to a *normalization factor*.

## Results

The hybrid system combines the regular expression classifier with the vector-space classifier. Unlike [6] we do not merge our classifiers by linear combination because the RegEx module does not return a scoring consistent with the vector space system. Therefore, the combination does not use the RegEx's score. Instead, it uses the list returned by the vector space module as a *reference *list (*RL*), while the list returned by the regular expression module is used as *boosting *list (*BL*) which serves in order to improve the ranking of terms listed in *RL*. A third factor takes into account the length of terms: both the term's length measured in words (*L*_1_) and the character length of terms (*L*_2_, with *L*_2 _> 3) are computed, so that long and/or multi-word terms appearing in both lists are favored over short and/or single word terms. We assume that the reference list has a good recall, and we do not set any threshold on it. For each term *t *listed in the *RL*, the combined Retrieval Status Value (RSV) is:

(1) $$RSV_{Hybrid}=\{\begin{array}{ll} RSV_{VS}(t)\cdot Ln(L_{1}(t)\cdot L_{2}(t)\cdot k) & \text{if }t\in BL,\\ RSV_{VS}(t) & \text{otherwise}. \end{array}$$

The k parameter is set empirically using the tuning data. Table 2 shows that the optimal tf.idf parameters *lnc.atn *for the basic VS classifier does not provide the optimal combination with RegEx. The optimal combination is obtained with *ltc.lnn *settings (see [16] for a comprehensive presentation of the mathematical formulae supporting the tf.idf weighting schemas). We also observe that the *atn.ntn *weighting schema maximizes the top candidate (i.e. *Precision*_*at Recall *= 0_) measure, but for a general purpose system, we prefer to maximize average precision, since this is the only measure that summarizes the performance of the full ordering of concepts. However, in the context of a fully automatic system, the top-ranked concepts are clearly of major importance, therefore we also provide this measure.

**Table 2:** Results of the system when combining the vector space and the regular expression modules.

Weighting function concepts.abstracts	Top Precision	Mean average Precision
Hybrids: tf.idf (VS) + RegEx		

ltc.lnn	0.800	0.4545
lnc.lnn	0.791	0.453
anc.ntn	0.787	0.4515
atn.ntn	0.823	0.4485

The top precision (82.3%) is in the range of what has been reported elsewhere [17,18], while the search space of our tool (800 000 terms and 1000 documents) is much larger than in these experiments which work with some hundreds categories and use sentences rather than abstracts for the categorization. Such a precision means that the top-returned category is one of the expected categories in 8 cases out of 10. The measured mean average precision of almost 50% (0.45%) means that half of the expected categories are proposed by the system.

## Qualitative evaluation and discussion

To conduct the qualitative evaluation, we looked at a sample of twelve abstracts. A unique judge manually controlled the top three categories provided by the SNOMED categorizer to find if there were non-MeSH categories which could have been relevant for indexing the abstract. For eight abstracts, a relevant category was found in the SNOMED ranking. A typical example is given in Figures 1 and 2. Thus, the SNOMED terminology contains the concept *glucuronic acid*, which could have been chosen to index the content of the article based on its abstract, but the concept does not exist in the MeSH. We also can observe that the current setting of the system, which favors exact match concepts (via the regular expression module) and content-bearing features (via the document frequency factor), seems somehow able to discard some very lengthy SNOMED concepts such as *nontraffic accident involving collision of motor-driven snow vehicle, not on public highway, driver of motor vehicle injured*. This qualitative observation suggests that the conceptual coverage of SNOMED can be larger than the MeSH and that automatic indexing could be improved regarding recall by using at least some SNOMED codes. This observation must be balanced by our preliminary remarks: several MeSH categories cannot be appropriately mapped into SNOMED CT.

More generally, we believe that the size and complexity of advanced ontological systems such as SNOMED do demand the development of specific computer tools to make possible their manipulation by real users, including professional encoders. Indeed, given the low rate of coding agreement reported when using large terminologies, e.g. [19], which at best represent only a fraction of SNOMED CT regarding their size and complexity, it is expected that large-scale ontology-driven coding tasks will require computer tools to assist users for maintaining and operating. Unfortunately, we observe that the research in the field seem to concentrate most of efforts on clinical and/or formal issues, whereas access, search and navigation capabilities tend to receive a fairly limited interest.

## Conclusion and future work

We have reported on the development and preliminary evaluation of a new type of categorization and browsing tools for SNOMED encoding. The system combines a pattern matcher, based on regular expressions of terms, and a vector space retrieval engine that uses stems as indexing terms, a traditional *tf.idf *weighting schema, and cosine as normalization factor. For top returned concepts, a precision of more than 80% is observed, which seems sufficient to help coding textual contents with SNOMED categories. A manual and qualitative evaluation on a dozen of MEDLINE abstracts suggests that SNOMED CT could represent an improvement compared to existing broad medical terminologies such as the MeSH. Clearly, further studies will be needed using clinical cases directly annotated with SNOMED categories, as described in [17] and [20]. Usability studies [21] are also needed to assess the relevance of the text-to-SNOMED associations provided by the tool from a coder perspective. Furthermore, from encoding and billing perspectives, SNOMED codes should be mapped to the International Classification of Disease and/or to Diagnosis Related Groups [22] using an unambiguous model [23,24] to evaluate the appropriateness of the SNOMED CT encoding for monitoring health systems.

## Competing interests

The authors declare that they have no competing interests.

## Authors' contributions

The first two authors: design and evaluation of the tools. The other two authors: determination of the user requirements, and test of the graphic user interface.

## References

[B1] Ehrler F, Jimeno A, Geissbühler A, Ruch P (2005). Data-poor Categorization and Passage Retrieval for Gene Ontology Annotation in Swiss-Prot. BMC Bioinformatics.

[B2] Gobeill J, Tbahriti I, Ehrler F, Mottaz A, Veuthey A, Ruch P (2008). Gene Ontology density estimation and discourse analysis for automatic GeneRiF extraction. BMC Bioinformatics.

[B3] Aronson A, Demner-Fushman D, Humphrey S, Lin J, Liu H, Ruch P, Ruiz M, Smith L, Tanabe L, Wilbur J (2006). Fusion of Knowledge-intensive and Statistical Approaches for Retrieving and Annotating Textual Genomics Documents. TREC 2005.

[B4] Shaw W, Wood J, Wood R, Tibbo H (1991). The Cystic Fibrosis Database: Content and Research Opportunities. LSIR.

[B5] Marti Hearst's pages. http://www.sims.berkeley.edu/~hearst/irbook/.

[B6] Larkey L, Croft W (1996). Combining classifiers in text categorization. SIGIR.

[B7] Manber U, Wu S (1994). GLIMPSE: A Tool to Search Through Entire File Systems. Proceedings of the USENIX Winter 1994 Technical Conference, San Francisco CA USA.

[B8] Ruch P (2002). Using contextual spelling correction to improve retrieval effectiveness in degraded text collections. COLING 2002.

[B9] Aronson A, Bodenreider O, Chang H, Humphrey S, Mork J, Nelson S, Rindflesch T, Wilbur W (1999). The Indexing Initiative. A Report to the Board of Scientific Counselors of the Lister Hill National Center for Biomedical Communications. Tech rep, NLM.

[B10] Ruch P, Baud R, Geissbühler A (2003). Learning-Free Text Categorization. LNAI 2780.

[B11] Ruch P (2006). Automatic Assignment of Biomedical Categories: Toward a Generic Approach. Bioinformatics.

[B12] Hull D (1996). Stemming Algorithms: A Case Study for Detailed Evaluation. Journal of the American Society of Information Science.

[B13] Baud R, Nystrom M, Borin L, Evans R, Schulz S, Zweigenbaum P (2005). Interchanging Lexical Information for a Multilingual Dictionary. AMIA Symposium Proceedings.

[B14] Ruch P (2004). Query Translation by Text Categorization. COLING 2004.

[B15] Singhal A, Buckley C, Mitra M (1996). Pivoted document length normalization. ACM-SIGIR.

[B16] Ruch P, Ehrler F, Abdou S, Savoy J (2006). Report on the TREC2005 Experiment: Genomics Track. TREC 2005.

[B17] Friedman C, Shagina L, Lussier Y, Hripcsak G (2004). Automated encoding of clinical documents based on natural language processing. J Am Med Inform Assoc.

[B18] Lussier Y, Shagina L, Friedman C (2001). Automating SNOMED coding using medical language understanding: a feasibility study. J Am Med Inform Assoc (Symposium Suppl).

[B19] Funk M, Reid C (1983). Indexing consistency in MEDLINE. Bull Med Libr Assoc.

[B20] de Bruijn L, Hasman A, Arends J (1999). Automatic SNOMED classification – a corpus based method. Yearbook of Medical Informatics.

[B21] Despont-Gros C, Mueller H, Lovis C (2005). Evaluating user interactions with clinical information systems: a model based on human-computer interaction models. J Biomed Inform.

[B22] Bowman S (2005). Coordinating SNOMED-CT and ICD-10. J AHIMA.

[B23] Rassinoux A, Baud R, Ruch P, Trombert-Paviot B, Rodrigues J (1999). Model-based Semantic Dictionaries for Medical Language Understanding. J Am Med Inform Assoc (Symp Suppl).

[B24] Rodrigues J, Rector A, Zanstra P, R RB, Innes K, Rogers J, Rassinoux A, Schulz S, Paviot BT, ten Napel H, Clavel L, Haring E van der, Mateus C (2006). An Ontology driven collaborative development for biomedical terminologies: from the French CCAM to the Australian ICHI coding system. Stud Health Technol Inform.

